# Serum metabolomic profiling of exercise response and hyperuricemia status in athletes: a two-part exploratory study

**DOI:** 10.1186/s13102-026-01907-2

**Published:** 2026-07-31

**Authors:** Siqi Yu, Liju Fan, Xiaochen Huang, Xiaoqing Xing, Lili Xing, Jing An, Guannan Zhang

**Affiliations:** 1 School of Pharmacy, Hebei Medical University, Shijiazhuang, Hebei 050017 P.R. China; 2 Pharmaceutical department, Hebei General Hospital, Shijiazhuang, Hebei 050051 P.R. China; 3 Hebei Institute of Sports Science, Shijiazhuang, Hebei 050000 P.R. China; 4 Hebei Key Laboratory of Digital Physical Fitness Monitoring and Health Promotion, Shijiazhuang, Hebei 050000 P.R. China

**Keywords:** High-intensity exercise, Hyperuricemia, Metabolomics, Lysophosphatidylcholine, Lipid metabolism

## Abstract

**Background:**

While high-intensity training improves physiological functions, it also induces significant metabolic stress, leading to a higher incidence of hyperuricemia among athletes compared to the general population. However, existing metabolomic studies on the association between high-intensity exercise and hyperuricemia remain limited, lacking mechanistic exploration of the link between acute exercise stress and chronic disease risk. This study aimed to investigate the potential metabolic associations between acute exercise stress and chronic hyperuricemia through using untargeted metabolomics, while identifying disease-related biomarkers.

**Methods:**

This study employed LC-MS-based untargeted metabolomics. First, a self-controlled intervention was conducted in 41 normouricemic judo athletes to analyze immediate changes in serum metabolites before and immediately after high-intensity resistance exercise. Subsequently, a validation study was performed in a specific cohort to compare the resting metabolomic profiles between athletes with hyperuricemia and normouricemic controls.

**Results:**

High-intensity resistance exercise induced significant alterations primarily in glycerophospholipid metabolism. Specifically, lysophosphatidylcholines (LysoPC) were markedly upregulated, whereas phosphatidylcholines (PC) were significantly downregulated immediately after exercise. LysoPC levels showed significant correlations with multiple clinical indices, and a subsequent comparative analysis between hyperuricemic and normouricemic athletes further identified differential metabolites closely associated with hyperuricemia risk.

**Conclusions:**

This study explored the potential metabolic associations between acute exercise stress and chronic hyperuricemia. Key lipid metabolites, represented by LysoPC, hold promise as potential biomarkers. However, owing to the absence of recovery-phase data, the long-term relationship between these transient post-exercise changes and chronic hyperuricemia requires further validation.

**Trial registration:**

Chinese Clinical Trial Registry (ChiCTR), ChiCTR2600126268. Registered 5 June 2026 (retrospectively registered).

## Introduction

While high-intensity exercise enhances physiological function, the associated stress profoundly influences metabolic homeostasis [1]. Regulation of uric acid metabolism in athletes is a critical focus in exercise physiology and sports medicine, particularly because individuals engaged in high-intensity training exhibit a higher prevalence of hyperuricemia and gout than the general population [2].

Prolonged high-intensity exercise increases oxidative stress biomarkers in blood and skeletal muscle [3], potentially impairing energy metabolism homeostasis. The demarcation between exercise-induced health adaptations and the potential risks arising from metabolic stress remain poorly defined, hindering the development of clear guidelines for optimal exercise limits [4]. Moderate exercise can reduce the risk of chronic metabolic diseases, improving metabolic function in organs such as the liver, adipose tissue, vasculature, and pancreas [5]. Consequently, clinical guidelines for managing hyperuricemia or gout universally endorse exercise as a crucial nonpharmacological intervention [6]. The precise assessment of individualized exercise load remains a persistent challenge in contemporary sports science [7]. When exercise intensity exceeds physiological tolerance, excessive stress responses may disrupt metabolic pathways, including enhanced glycolysis leading to lactate accumulation [5] or aberrant lipid catabolism causing altered free fatty acid levels [8]. These individualized differences in metabolic regulation create a complex dynamic between the beneficial and adverse effects of exercise and highlight the need for effective strategies to prevent and manage exercise-related metabolic disorders.

Metabolomics has emerged as a powerful tool for elucidating the relationship between physical activity and systemic metabolism [9]. However, existing studies are often confined to measuring metabolic biomarkers at single time points [10] or focus solely on metabolic changes immediately before and after exercise [11], without integrating acute exercise-induced metabolic responses with profiles associated with chronic hyperuricemia. The concept of metabolic flexibility, defined as the capacity to respond or adapt to changes in metabolic or energetic demand in response to environmental or activity-related stimuli [12], has gained traction recently. Currently, metabolomics-based evidence remains limited regarding whether metabolic flexibility can predict the transition from acute metabolic perturbations induced by high-intensity exercise to chronic metabolic risk.

In this study, active judo athletes were selected as the study population, and heavy resistance training was chosen as the exercise modality. Heavy resistance training is a core and frequently performed component of judo training, typically conducted approximately three times per week, which makes this protocol ecologically relevant to the target population. In addition, previous studies have shown that resistance training may lead to increased purine catabolism and lactate accumulation [13], which are physiologically relevant to uric acid metabolism. Therefore, compared with endurance-based exercise modalities, heavy resistance training represents a sport-specific and physiologically relevant exercise stressor for exploring metabolic perturbations associated with uric acid regulation in judo athletes. The protocol used in this study was derived from conventional judo strength training sessions and included clearly controllable parameters, including exercise type, load, sets, repetitions, and rest intervals, thereby improving reproducibility in future validation studies.

This study aimed to explore the metabolic correlation between exercise-induced metabolic stress and hyperuricemia. First, serum samples were collected from athletes before and after high-intensity resistance exercise, and metabolomics was performed to identify differentially expressed metabolites and associated pathways, with a focus on purine metabolism, energy metabolism, and oxidative stress. Second, a cross-sectional validation compared resting metabolic profiles between athletes with hyperuricemia and those with normal uric acid levels. This approach sought to identify metabolic pathways and specific metabolites associated with uric acid dysregulation. By integrating these metabolomic findings, we aimed to uncover potential mechanistic links between acute exercise stress and the development of chronic disease. This approach may facilitate the identification of metabolic indicators associated with emerging health risks in athletes, ultimately contributing to the preservation of their long-term health and the sustainability of their competitive careers.

## Methods

### Study participants

Sixty-five athletes (33 males, 32 females; age range: 20–26 years; ≥7 years of training experience) were recruited and allocated to two study groups: (i) a self-controlled intervention group examining metabolic changes before and after high-intensity resistance exercise in normouricemic athletes, and (ii) a cross-sectional validation group comparing resting metabolomic profiles between hyperuricemic and normouricemic athletes. All participants were fully informed of the study purpose, procedures, and potential risks and provided written informed consent. The study protocol was approved by the Ethics Review Committee (Approval No. LL2025002).

#### Inclusion criteria

Participants were aged ≥ 18 years, had no major musculoskeletal injuries or acute illnesses in the past month, and were not taking medications or nutritional supplements during this period.

#### Group-specific criteria

Based on the general inclusion criteria, participants were categorized as follows: Hyperuricemic group: Athletes met the diagnostic criteria for hyperuricemia, defined as fasting serum uric acid levels > 420 µmol/L (7.0 mg/dL) on two separate occasions under a normal purine diet [14]. Normouricemic control group: Athletes had normal serum uric acid levels (< 420 µmol/L) and were matched to the hyperuricemic group based on age and training duration.

#### Exclusion criteria

Athletes with cardiac, hepatic, or renal disease; diabetes mellitus or other metabolic disorders; or musculoskeletal disorders. Female athletes who were menstruating, pregnant, or lactating.

### Materials

Thermo Scientific™ Orbitrap Exploris 240 high-resolution mass spectrometer (Thermo Fisher Scientific, USA) equipped with an electrospray ionization (ESI) source; ultrasonic cleaner S-450 H (Elma, Germany); vortex mixer (Vortex Genie-2; Scientific Industries, USA); multitube vortex mixer (Multi Reax; Heidolph, Germany); high-speed refrigerated centrifuge (CT15RT; Shanghai Tianmei, China); ultrapure water system (Milli-Q; Millipore, USA).

Methanol and acetonitrile (HPLC grade; Thermo Fisher Scientific); formic acid (HPLC grade; Shanghai Macklin Biochemical Technology).

### Methods

#### Experimental design

Part 1: Self-controlled intervention in athletes before and after high-intensity resistance exercise. Athletes performed a single session of high-intensity resistance exercise (detailed protocol in High-intensity resistance exercise protocol section). Blood samples were collected at baseline (pre-exercise) and immediately after exercise (within 10 min after completion) [15]. Metabolomic profiles were obtained at both time points, and metabolic signatures were compared before and after exercise in the 41 enrolled athletes(19 male athletes, 22 female athletes).

Part 2: Cross-sectional validation. Compared the resting metabolic profiles of athletes with hyperuricemia (*n* = 8, 5 male athletes, 3 female athletes) with a control group with normal uric acid levels (*n* = 16, 9 male athletes, 7 female athletes).

#### Sample collection and pretreatment

Sample collection: All athletes refrained from strenuous exercise for 48 h before testing and followed a standardized diet. To control for confounding dietary factors, athletes avoided alcohol, sugary drinks, and high-purine foods for 3 days prior. All meals were provided by the team cafeteria, with daily dietary records kept by the nutritionist to ensure stable purine intake. A 24-hour dietary recall was administered on the day prior to the exercise intervention [16]. Dietary information was collected by the research team, and total purine intake was estimated using the “Dietary Nutrition Analysis and Management Software for Athletes and Fitness Populations” (developed by Institute of Sports Medicine, General Administration of Sport of China). All participants’ purine intake was strictly controlled within a normal range (200–600 mg/day) with no statistical differences between groups [17]. Furthermore, to avoid the compounding stress of fasting-induced lipid mobilization, participants were not in a fasting state during the exercise. They consumed a standardized breakfast at 7:30−8:00 AM (breakfast consisted of a standard meal containing complex carbohydrates, high-quality protein, vegetables, and a small amount of fat). The exercise commenced at 9:30 AM (1.5–2 h post-meal). Baseline pre-exercise blood samples were collected in the morning, and post-exercise samples were collected within 10 min after completion of the high-intensity heavy resistance training session. For the validation group, fasting blood samples were collected in the morning during a recovery period (at least 48 h after the last strenuous exercise).The blood collection tube type is a standard tube without anticoagulant.

Sample pretreatment: Serum (100 µL) was aliquoted, and 400 µL of acetonitrile: methanol (1:1, v/v) was added. The mixture was vortexed for 30 s, sonicated at 4 °C for 10 min, and then stored at −20 °C for 1 h. Samples were then centrifuged at 13,000 rpm for 15 min at 4 °C, and the supernatant was evaporated to dryness under nitrogen. The dried residue was reconstituted in 100 µL of acetonitrile: water (1:1, v/v), vortexed for 30 s, sonicated at 4 °C for 10 min, and centrifuged at 13,000 rpm for 15 min at 4 °C. The supernatant was collected for liquid chromatography-mass spectrometry analysis.

#### Chromatographic conditions

Mobile phase A consisted of 0.1% formic acid in water, and mobile phase B consisted of acetonitrile. The gradient elution program was as follows: 0–2 min, 5% B; 2–14 min, 5%–98% B; 14–20 min, 98% B; 20.1–25 min, 98%–5% B. The column temperature was maintained at 35 °C. Chromatographic column: Thermo Fisher Hypersil GOLD™ C18 (100 mm × 2.1 mm, 1.9 μm ).

#### Mass spectrometry conditions

Ion source: ESI operated in positive ion mode. Scan range: m/z 100–1500 Da.MS acquisition mode: Full MS-dd MS2 (data-dependent acquisition). Full MS resolution: 60,000; dd MS2 resolution: 15,000.Ion transfer tube temperature: 325 °C; vaporizer temperature: 350 °C; electrospray voltage: +3.5 kV in positive-ion mode.

#### Metabolomics analysis

Metabolomics data were acquired using the Thermo Scientific™ Orbitrap Exploris 240 high-resolution mass spectrometer and processed with Progenesis QI software for peak detection, alignment, and normalization.

Metabolites were identified by comparison with the Human Metabolome Database (HMDB), with a mass error tolerance of < 10 ppm, and further validated with reference to MS/MS fragmentation patterns.

#### High-intensity resistance exercise protocol

The high-intensity resistance training protocol used in this study was a sport-specific strength training regimen commonly employed in judo. The main training content included bench press, squat, bench row and circuit strength training. The exercise type, load intensity, number of sets, number of repetitions, rest scheme was formulated by the coaching team. Total training duration was approximately 2 h, including approximately 20 min of warm-up, 80 min of main training, and 20 min of cool-down (stretching and relaxation). Exercise intensity was assessed using the Borg 6–20 Rating of Perceived Exertion (RPE) scale, with a threshold of ≥ 15 (“hard” to “very hard”) defining high intensity. The actual mean RPE was 17 (“very hard”), confirming that the training met the criteria for high-intensity resistance exercise [18]. Detailed parameters for each exercise are provided in Table 1.

**Table 1:** High-intensity resistance exercise intervention protocol

Exercise	Load intensity (%1RM)	Actual load/weight	Sets	Repetitions per set	Rest between sets
Bench press	70%-90% 1RM	Starting at 90 kg, increasing 10–20 kg per set	5	8 − 3 (decreasing as load increases)	150–240 s (increasing as load increases)
Squat	70%-90% 1RM	Starting at 110 kg, increasing 10–20 kg per set	5	8 − 3 (decreasing as load increases)	150–240 s (increasing as load increases)
Bench Row	70%-90% 1RM	Starting at 80 kg, increasing 10–20 kg per set	5	8 − 2 (decreasing as load increases)	150–240 s (increasing as load increases)
Circuit 1: Barbell power clean	Fixed absolute weight	50 kg	4 (1 per circuit)	5	No rest between circuits
Circuit 2: Shoulder-mounted pull strap	Technical movement	Band resistance	4	10	No rest between circuits
Circuit 3: Dumbbell horizontal push	Fixed absolute weight	15 kg per side	4	10	No rest between circuits
Circuit 4: Power bike	Absolute resistance	300 watts	4	15 s all-out cycling	No rest between circuits
Circuit 5: Overhead barbell plate raise	Fixed absolute weight	25 kg	4	8	No rest between circuits
Circuit 6: Kettlebell farmer‘s walk	Fixed absolute weight	40 kg per side	4	15 m round trip	No rest between circuits

The circuit training portion (from power clean to farmer’s walk) was completed for 3 circuits. In each circuit, the 6 exercises were performed consecutively, with each exercise required to be completed within 15 s. There was no rest between exercises within a circuit, and 2 min of rest were given after each circuit

### Statistical analysis

Raw mass spectrometry data (RAW format) were imported into Progenesis QI for preprocessing and then subjected to multivariate statistical analysis using SIMCA 14.1 software platform.

Unsupervised principal component analysis (PCA) was performed to assess the overall distribution characteristics of samples and the stability of the analytical process. Orthogonal partial least squares discriminant analysis (OPLS-DA) was applied to maximize intergroup differences. To prevent overfitting of the OPLS-DA model, a 200-iteration permutation test was conducted to evaluate model validity.

The contribution of each metabolite to sample classification was assessed using the variable importance in projection (VIP) score derived from the OPLS-DA model, combined with q-value corrected by the Benjamini-Hochberg method. Metabolites with VIP > 1 from the first principal component of the OPLS-DA model and q-value corrected by the Benjamini-Hochberg method < 0.05 were considered significantly differential [19]. Identities of the selected differentially expressed metabolites were confirmed by querying the HMDB. Finally, based on the identified differential small-molecule metabolites, metabolic pathway enrichment analysis was performed using the MetaboAnalyst 6.0 platform and the Kyoto Encyclopedia of Genes and Genomes database. The enrichment ratio, calculated as the number of differentially expressed metabolites divided by the total number of detected metabolites in a given pathway, was used to quantify the degree of pathway enrichment. The P-value was used to assess the statistical significance of enrichment, with values closer to 0 indicating higher enrichment significance [19].

When analyzing the baseline data of the subjects, continuous variables were assessed for normality. Normally distributed data are presented as mean ± standard deviation (SD), while non-normally distributed data are expressed as median (interquartile range, P25-P75). Categorical variables are presented as frequencies (percentages).

## Results

### Exercise-induced alterations in the serum metabolome: acute response to high-intensity resistance training

#### Baseline characteristics

The baseline demographic and clinical characteristics of the athletes participating in the acute high-intensity resistance exercise intervention are summarized in Table 2.

**Table 2:** Baseline demographic and clinical characteristics of athletes

Characteristics	Total Participants (*n* = 41)
Sex (Male), n (%)	19 (46.3%)
Age (years)	22.0 (21.0, 25.0)
Height (cm)	173.1 ± 8.120
Weight (kg)	69.85(63.50,84.88)
Uric acid (µmol/L)	313.36 ± 84.11
Urea (mmol/L)	5.08 ± 1.01
Smoking (Yes), n (%)	0 (0%)
Drinking (Yes), n (%)	0 (0%)

Data are presented as mean ± SD or median (P25, P75). Normally distributed variables are expressed as mean ± SD, and non-normally distributed variables as median (P25, P75). None of the participants reported smoking or drinking

#### Multivariate statistical analysis

**Fig. 1:**
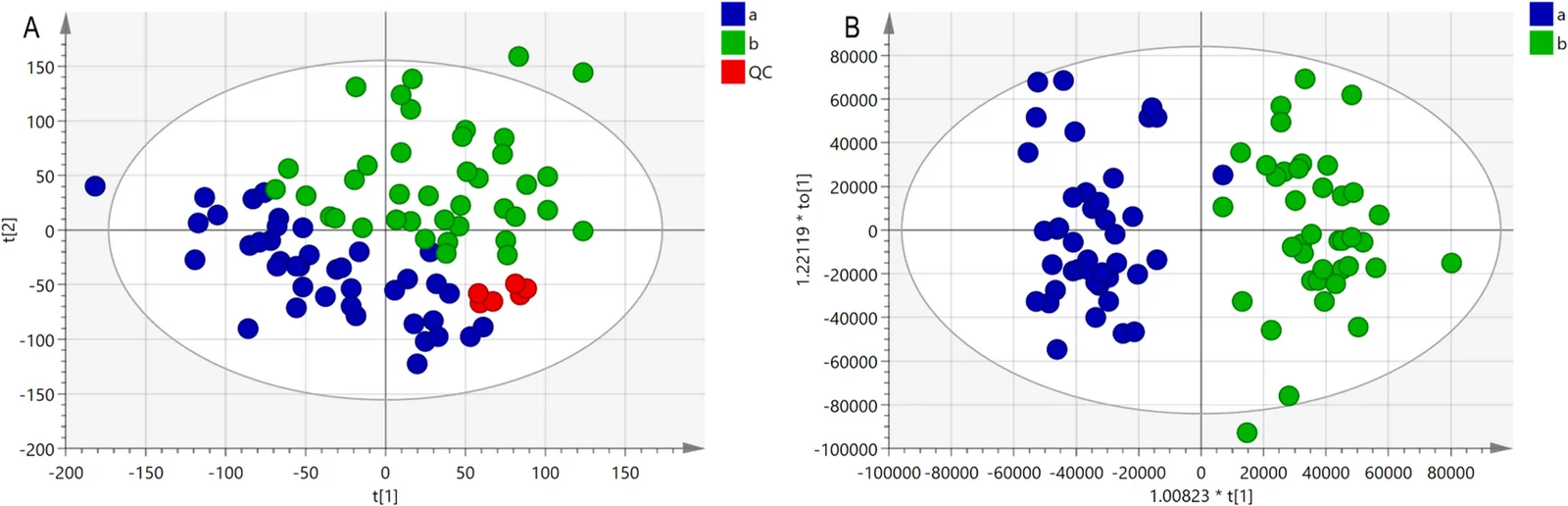
Multivariate statistical analysis of serum samples from athletes before and after high-intensity resistance exercise (A. PCA, B. OPLS-DA; (**a**) baseline, (**b**) post-exercise, QC. quality control)

PCA of the data acquired in positive ion mode revealed a clear separation trend between baseline and post-exercise samples, with quality control (QC) samples clustering tightly (Fig. 1A). This indicates distinct differences in metabolite composition and concentration between the two conditions. Most samples fell within the 95% confidence interval.

OPLS-DA demonstrated clear separation between the two groups, confirming the validity of the model (Fig. 1B). A 200-iteration permutation test (see Supplementary Material) showed no evidence of overfitting, with R² and Q² values positioned at the extreme right and the Q² regression line intersecting the y‑axis below zero.

#### Identification of differentially expressed metabolites

Differentially expressed metabolites were selected based on a VIP score > 1 from the OPLS-DA model and a q-value corrected by the Benjamini-Hochberg method < 0.05. Metabolite structures and molecular formulae were confirmed by matching MS/MS fragmentation patterns to the HMDB database. A total of 56 differentially expressed metabolites were identified following high-intensity resistance exercise intervention. Following the exclusion of exogenous compounds, 37 metabolites were retained for further analysis (Table 3).

**Table 3:** Differentially expressed metabolites from athletes before and after high-intensity resistance exercise

Differentially Expressed Metabolites	Retention Time	Molecular formula
Lumirubin	9.39_584.2630n	C_33_H_36_N_4_O_6_
7-[(1R,2R,3R)−3-Hydroxy−2-[(3 S)−3-hydroxyoct−1-enyl]−5-oxocyclopentyl]−5-heptenoic acid	8.79_352.2249n	C_20_H_32_O_5_
LysoPA(20:4(8Z,11Z,14Z,17Z)/0:0)	12.16_458.2433n	C_23_H_39_O_7_P
LysoPC(17:0/0:0)	13.27_509.3478n	C_25_H_52_NO_7_P
Phenylalanylphenylalanine	6.80_312.1472n	C_18_H_20_N_2_O_3_
11-Hydroxyoctadecanoylcarnitine	13.79_443.3608n	C_25_H_49_NO_5_
LysoPC(18:0/0:0)	13.93_523.3624n	C_26_H_54_NO_7_P
13-HODE	16.34_296.2350n	C_18_H_32_O_3_
3-Oxooctadecanoic acid	17.28_298.2507n	C_18_H_34_O_3_
(+)−15,16-Dihydroxyoctadecanoic acid	16.40_316.2612n	C_18_H_36_O_4_
Octanoylcarnitine	8.62_288.2168 m/z	C_15_H_29_NO_4_
6-Methylnonanoylcarnitine	9.64_316.2480 m/z	C_17_H_33_NO_4_
6-Dodecenoylcarnitine	10.68_342.2637 m/z	C_19_H_35_NO_4_
(2E,4E)-Tetradecadienoylcarnitine	11.01_368.2792 m/z	C_21_H_37_NO_4_
Tridec−4-enoylcarnitine	11.04_356.2794 m/z	C_20_H_37_NO_4_
10-methyltridecanoylcarnitine	12.35_372.3106 m/z	C_21_H_41_NO_4_
4-Methoxy−3-geranylgeranyl−1,2-dihydroxybenzene	14.75_413.3049 m/z	C_27_H_40_O_3_
12-Methylheptadecanoylcarnitine	14.90_428.3733 m/z	C_25_H_49_NO_4_
N-Nervonoyl Serine	15.15_454.3889 m/z	C_27_H_51_NO_4_
PC(18:3(9Z,12Z,15Z)/20:5(5Z,8Z,11Z,14Z,17Z))	15.07_802.5370 m/z	C_46_H_76_NO_8_P
4-Hexadecenoylcarnitine	12.69_398.3260 m/z	C_23_H_43_NO_4_
O-Palmitoylcarnitine	13.54_400.3419 m/z	C_23_H_45_NO_4_
Icosa−5,8,11-trienoylcarnitine	13.35_450.3577 m/z	C_27_H_47_NO_4_
LysoPS(18:0/0:0)	13.00_526.3145 m/z	C_24_H_48_NO_9_P
8-Methyltetradecanoylcarnitine	12.66_386.3267 m/z	C_22_H_43_NO_4_
LysoPC(*P*−16:0/0:0)	12.02_480.3446 m/z	C_24_H_50_NO_6_P
(8E,12Z)−10-Hydroxyoctadeca−8,12-dienoylcarnitine	12.31_422.3263 m/z	C_25_H_45_NO_5_
LysoPC(20:4(5Z,8Z,11Z,14Z)/0:0)	12.08_544.3388 m/z	C_28_H_50_NO_7_P
Myristoleoylcarnitine	12.00_370.2950 m/z	C_21_H_39_NO_4_
5-Methyldodecanoylcarnitine	11.52_358.2950 m/z	C_20_H_39_NO_4_
O-(17-Carboxyheptadecanoyl)carnitine	11.01_458.3473 m/z	C_25_H_47_NO_6_
3-Methylundecanoylcarnitine	10.86_344.2796 m/z	C_19_H_37_NO_4_
Undec−3-enoylcarnitine	9.84_328.2481 m/z	C_18_H_33_NO_4_
cis−4-Decenoyl carnitine	9.60_314.2325 m/z	C_17_H_31_NO_4_
(2E,6E,8E)-Decatrienoylcarnitine	8.65_310.2012 m/z	C_17_H_27_NO_4_
7-Hydroxyoctanoylcarnitine	7.05_304.2116 m/z	C_15_H_29_NO_5_
3-Octenoylcarnitine	7.83_286.2012 m/z	C_15_H_27_NO_4_

#### Pathway enrichment analysis

**Fig. 2:**
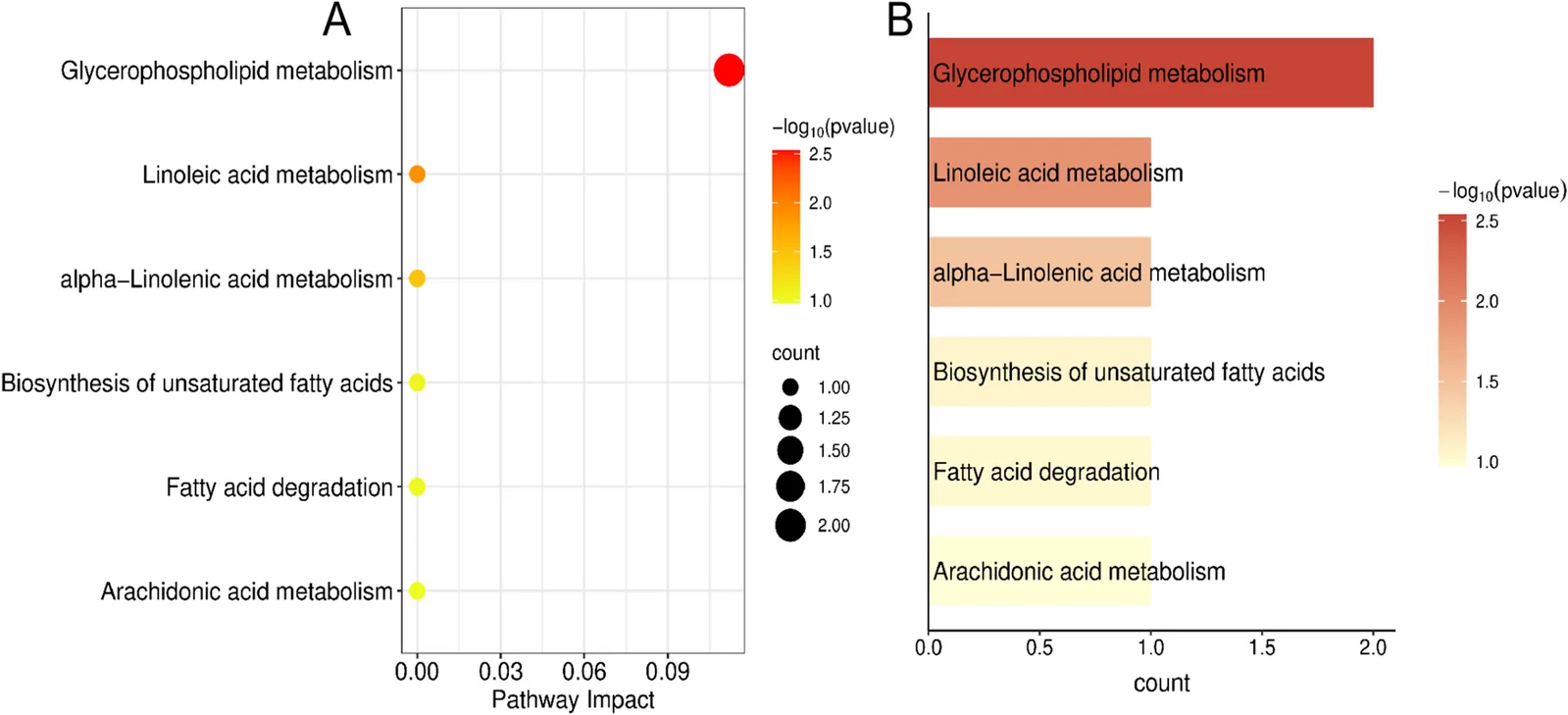
Pathway enrichment analysis before and after high-intensity resistance exercise (**A**. bar chart of pathway enrichment analysis before and after high-intensity resistance exercise, **B**. bubble chart of pathway enrichment analysis before and after high-intensity resistance exercise)

Pathway enrichment analysis identified six significantly enriched pathways (Fig. 2), including glycerophospholipid metabolism, linoleic acid metabolism, alpha-Linolenic acid metabolism, biosynthesis of unsaturated fatty acids, fatty acid degradation, aand rachidonic acid metabolism. The glycerophospholipid metabolism pathway showed the highest enrichment, which is associated with lipid metabolism. Lysophosphatidylcholine (LysoPC) and Phosphatidylcholine(PC) were identified as key metabolites contributing to enrichment in these pathways.

#### Hierarchical clustering heatmap analysis of differentially expressed metabolites

**Fig. 3:**
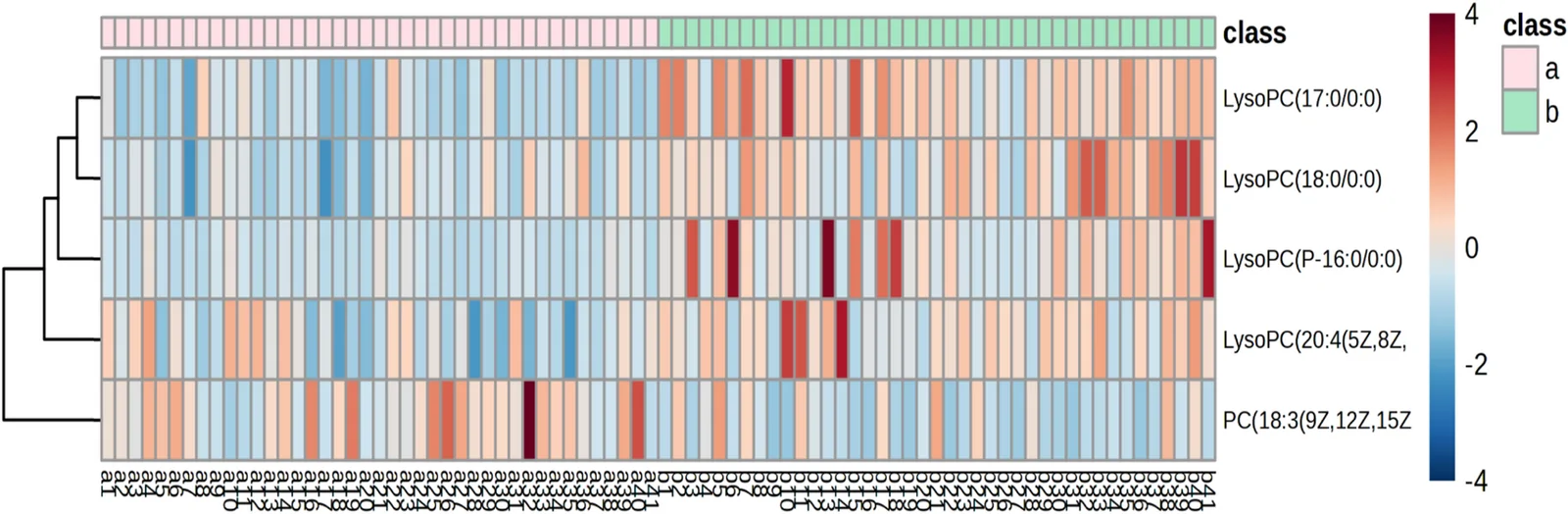
Heatmap of LysoPC and PC before and after high-intensity resistance exercise(a. baseline, b. post-exercise)

To visualize the differences between experimental conditions, we performed hierarchical clustering heatmap analysis of the relative intensities of differential serum metabolites using MetaboAnalyst 6.0. Compared to baseline, LysoPC were significantly upregulated immediately after exercise, the expression level of PC is significantly downregulated. (Fig. 3).

#### Correlation analysis between ΔLysoPC levels and clinical parameters

We employed two correlation analysis approaches to examine the associations between changes in LysoPC levels before and after exercise and a panel of post-exercise clinical parameters, including urea, creatine kinase (CK), cortisol (C), magnesium (Mg), sodium (Na), potassium (K), testosterone (T), hemoglobin (Hgb), hematocrit (Hct), uric acid (UA), calcium (Ca), albumin (ALB), immunoglobulin G (IGG), phosphorus (P), immunoglobulin M (IGM), chloride (Cl), glucose (GLU), immunoglobulin A (IGA), serum iron (Fe), total cholesterol (CHOL), triglycerides (TG), and white blood cell count (WBC).

**Fig. 4:**
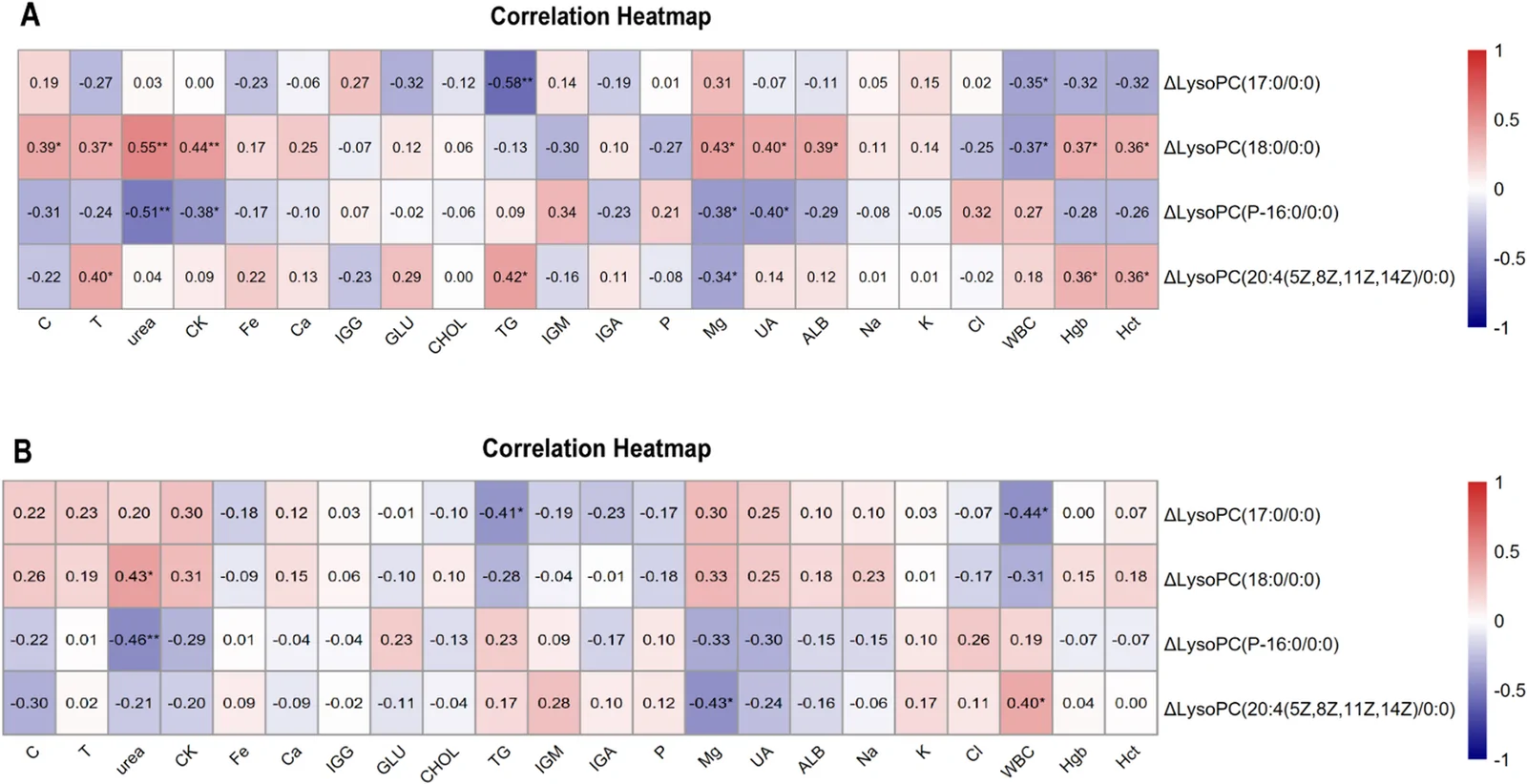
Correlation heatmaps showing associations between exercise-induced changes in LysoPC levels (ΔLysoPC) and clinical parameters after heavy resistance training. (**A**) Spearman correlation analysis without sex adjustment. (**B**) Partial correlation analysis adjusted for sex.

Before adjusting for sex, Spearman correlation analysis revealed that ΔLysoPC (17:0) was negatively correlated with TG and WBC; ΔLysoPC (18:0) was significantly negatively correlated with WBC and significantly positively correlated with T, urea, CK, P, Mg, UA, ALB, Hgb, and Hct; ΔLysoPC (P-16:0) was significantly negatively correlated with urea, CK, Mg, and UA; and ΔLysoPC (20:4) was significantly positively correlated with T, TG, Hgb, and Hct, and significantly negatively correlated with Mg (Fig. 4A).After applying partial correlation analysis to control for the effect of sex differences, ΔLysoPC (17:0) remained significantly negatively correlated with TG and WBC; ΔLysoPC (18:0) showed a significant positive correlation with urea; ΔLysoPC (P-16:0) exhibited a significant negative correlation with urea; and ΔLysoPC (20:4) was significantly negatively correlated with Mg and significantly positively correlated with WBC (Fig. 4B).These findings suggest that the associations between exercise-induced LysoPC changes and several post-exercise clinical parameters, particularly CK, UA, and hematological indices, may be partly influenced by sex-related differences.

### Comparative serum metabolomic profiling: hyperuricemic vs. normouricemic athletes at rest

#### Baseline characteristics

The baseline demographic and clinical characteristics of the hyperuricemic and normouricemic athletes are summarized in Table 4. Notably, while sex, age, height were comparable between groups, body weight was significantly higher in the hyperuricemic group, consistent with the known epidemiological association between obesity and hyperuricemia. And as expected, serum uric acid levels were significantly different (*p* < 0.001), confirming the successful grouping.

**Table 4:** Baseline demographic and clinical characteristics of athletes

Characteristics	Normal UA (*n* = 16)	High UA (*n* = 8)	*P*
Sex (Male), n (%)	9 (56.2%)	5 (62.5%)	1.000
Age (years)	24.0 (21.0, 26.0)	21.5 (21.0, 22.0)	0.094
Height (cm)	174.6 ± 9.4	179.4 ± 4.9	0.196
Weight (kg)	78.28 ± 19.69	109.76 ± 14.91	< 0.001*
Uric acid (µmol/L)	338.5 (209.8, 381.3)	476.0 (423.0, 513.5)	< 0.001*
Urea (mmol/L)	4.90(4.07,5.40)	5.76(4.31,6.92)	0.114
Smoking (Yes), n (%)	0(0%)	0(0%)	-
Drinking (Yes), n (%)	0(0%)	0(0%)	-

Data are presented as mean ± SD, median (P25, P75), or n (%). Between-group comparisons were performed using independent-samples t-test for normally distributed variables, Mann-Whitney U test for non-normally distributed variables, and Fisher’s exact test (two-tailed) for sex distribution (*P* = 1.000). Since none of the participants in either group reported smoking or drinking, statistical comparison was not performed (indicated as “-”). *P < 0.05 was considered statistically significant

#### Multivariate statistical analysis

**Fig. 5:**
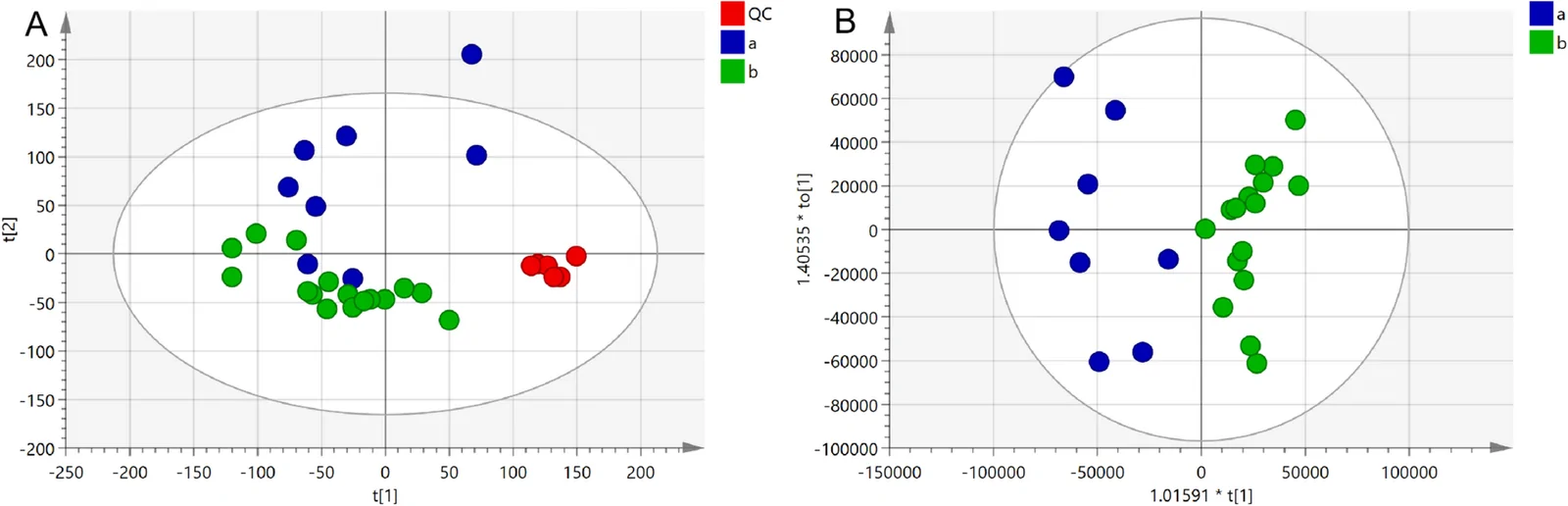
Multivariate statistical analysis of resting serum samples from hyperuricemic and normouricemic athletes (A. PCA, B. OPLS-DA; (**a**) hyperuricemic, (**b**) normouricemic, QC. quality control)

PCA of data acquired in positive ion mode revealed a clear separation trend between resting serum samples from hyperuricemic and normouricemic athletes, indicating differences in metabolite composition and concentration between the two groups (Fig. 5A). QC samples clustered tightly, indicating robust data quality. Most samples fell within the 95% confidence interval.

OPLS-DA demonstrated complete separation between groups, confirming the validity of the model (Fig. 5B). A 200-iteration permutation test (see Supplementary Material) showed no evidence of overfitting, with R² and Q² values positioned at the extreme right and the Q² regression line intersecting the y‑axis below zero.

#### Identification of differentially expressed metabolites

Comparative analysis between hyperuricemic and normouricemic athletes identified 80 metabolites with significantly different abundances. After excluding exogenous compounds, 47 metabolites were retained for further analysis (Table 5).

**Table 5:** Differentially expressed metabolites from hyperuricemic and normouricemic athletes

Differentially Expressed Metabolites	Retention Time	Molecular formula
(2,8)-Decadienoylcarnitine	8.93_312.2168 m/z	C_17_H_29_NO_4_
(25R)-3beta-hydroxycholest-5-en-7-one-26-oate	12.06_431.3150 m/z	C_27_H_42_O_4_
(2E,4E)-Tetradecadienoylcarnitine	11.01_368.2792 m/z	C_21_H_37_NO_4_
(2E,5Z,7E)-Decatrienoylcarnitine	8.54_310.2012 m/z	C_17_H_27_NO_4_
(2E,6E,8E)-Decatrienoylcarnitine	8.65_310.2012 m/z	C_17_H_27_NO_4_
(2Z)-Non-2-enoylcarnitine	8.65_300.2169 m/z	C_16_H_29_NO_4_
(3Z,5E,7E)-Decatrienoylcarnitine	8.25_310.2013 m/z	C_17_H_27_NO_4_
(5Z,7E)-9,10-Seco-5,7,10(19)-cholestatriene	20.72_369.3514 m/z	C_27_H_44_
10-methyltridecanoylcarnitine	12.35_372.3106 m/z	C_21_H_41_NO_4_
11-Hydroxyoctadecanoylcarnitine	13.79_443.3608n	C_25_H_49_NO_5_
12-Methylheptadecanoylcarnitine	14.90_428.3733 m/z	C_25_H_49_NO_4_
2-Pentenoic acid	7.64_101.0596 m/z	C_5_H_8_O_2_
3-hydroxydecanoyl carnitine	8.58_332.2431 m/z	C_17_H_33_NO_5_
3-Methylundecanoylcarnitine	10.86_344.2796 m/z	C_19_H_37_NO_4_
3-Octenoylcarnitine	7.83_286.2012 m/z	C_15_H_27_NO_4_
4alpha-Carboxy-5alpha-cholesta-8-en-3beta-ol	18.83_431.3518 m/z	C_28_H_46_O_3_
4-Methylpentanoylcarnitine	6.99_260.1854 m/z	C_13_H_25_NO_4_
5-Oxooctadecanoic acid	16.69_299.2579 m/z	C_18_H_34_O_3_
6-Dodecenoylcarnitine	10.68_342.2637 m/z	C_19_H_35_NO_4_
6-Methylnonanoylcarnitine	9.64_316.2480 m/z	C_17_H_33_NO_4_
7-Hydroxyoctanoylcarnitine	7.05_304.2116 m/z	C_15_H_29_NO_5_
8-Methyltetradecanoylcarnitine	12.66_386.3267 m/z	C_22_H_43_NO_4_
cis-4-Decenoyl carnitine	9.23_314.2326 m/z	C_17_H_31_NO_4_
DG(20:4(5Z,8Z,11Z,14Z)/18:2(9Z,12Z)/0:0)	20.24_658.5405 m/z	C_41_H_68_O_5_
Dihomo-gamma-linolenoylethanolamide	13.27_349.2980n	C_22_H_39_NO_2_
Dodecatrienoic acid	8.33_194.1306n	C_12_H_18_O_2_
GlcCer(d18:1/16:0)	19.02_699.5644n	C_40_H_77_NO_8_
Heptanoylcarnitine	7.52_274.2012 m/z	C_14_H_27_NO_4_
LacCer(d18:1/16:0)	18.58_861.6175n	C_46_H_87_NO_13_
Lumirubin	9.39_584.2630n	C_33_H_36_N_4_O_6_
LysoPA(0:0/18:0)	14.40_421.2712 m/z	C_21_H_43_O_7_P
LysoPA(20:4(8Z,11Z,14Z,17Z)/0:0)	12.16_458.2433n	C_23_H_39_O_7_P
LysoPC(17:0/0:0)	13.27_509.3478n	C_25_H_52_NO_7_P
LysoPC(18:0/0:0)	13.93_523.3624n	C_26_H_54_NO_7_P
LysoPC(20:4(5Z,8Z,11Z,14Z)/0:0)	12.08_544.3388 m/z	C_28_H_50_NO_7_P
LysoPC(22:5(4Z,7Z,10Z,13Z,16Z)/0:0)	12.16_570.3549 m/z	C_30_H_52_NO_7_P
Myristoleoylcarnitine	12.00_370.2950 m/z	C_21_H_39_NO_4_
O-(17-Carboxyheptadecanoyl)carnitine	11.01_458.3473 m/z	C_25_H_47_NO_6_
Octadeca-9,11-dienoic acid	15.31_280.2401n	C_18_H_32_O_2_
Octanoylcarnitine	8.62_288.2168 m/z	C_15_H_29_NO_4_
O-Palmitoylcarnitine	13.54_400.3419 m/z	C_23_H_45_NO_4_
PC(16:1(9Z)/22:6(4Z,7Z,10Z,13Z,16Z,19Z))	15.40_803.5437n	C_46_H_78_NO_8_P
PC(18:2(9Z,12Z)/15:0)	14.86_744.5551 m/z	C_41_H_78_NO_8_P
Phenylalanylphenylalanine	6.80_312.1472n	C_18_H_20_N_2_O_3_
Tridec-4-enoylcarnitine	11.04_356.2794 m/z	C_20_H_37_NO_4_
Undec-3-enoylcarnitine	9.84_328.2481 m/z	C_18_H_33_NO_4_
Uric acid	0.93_169.0354 m/z	C_5_H_4_N_4_O_3_

#### Pathway enrichment analysis

**Fig. 6:**
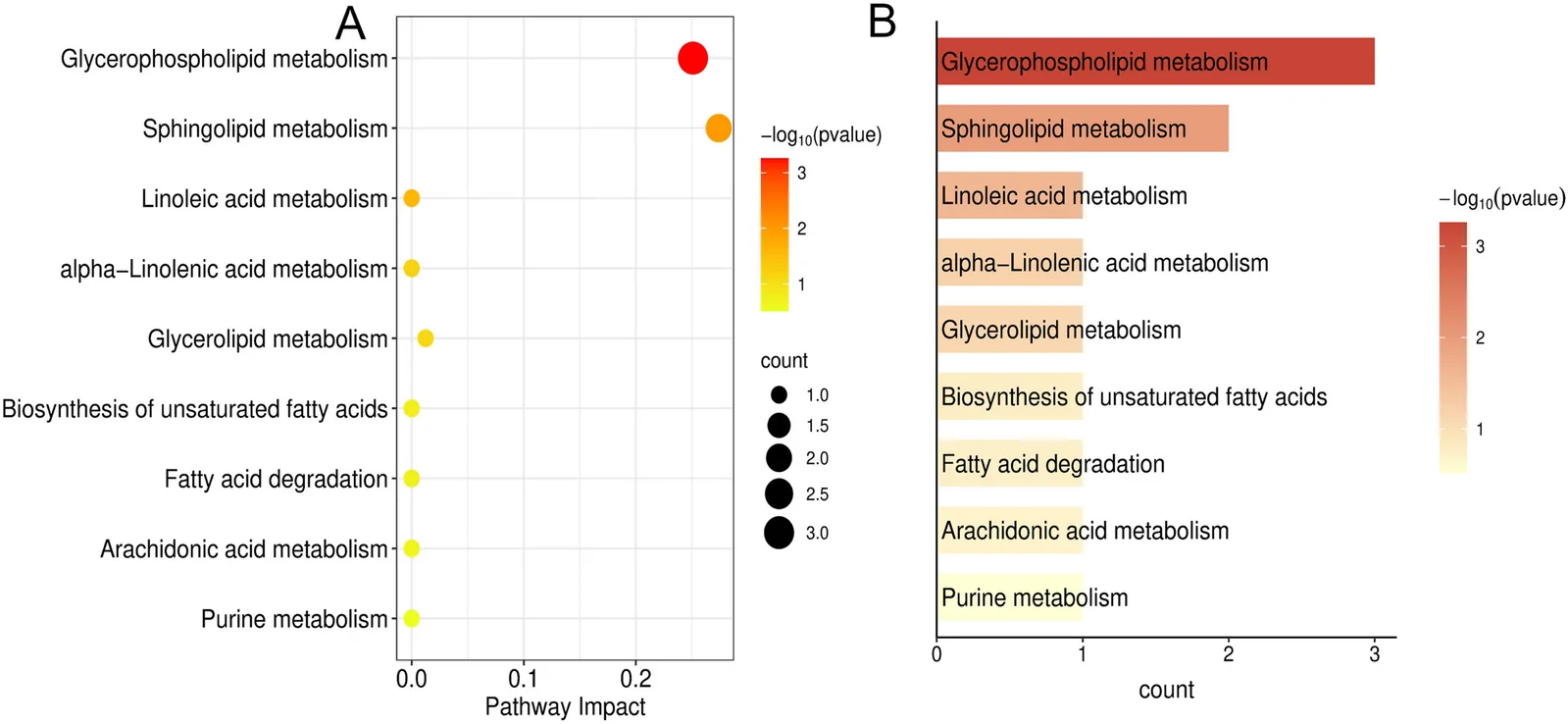
Pathway enrichment analysis in hyperuricemic vs. normouricemic athletes at rest (**A**. bar chart of pathway enrichment analysis in hyperuricemic vs. normouricemic athletes at rest, **B**. bubble chart of pathway enrichment analysis in hyperuricemic vs. normouricemic athletes at rest)

Pathway enrichment analysis of the differentially expressed metabolites revealed 9 significantly enriched metabolic pathways (Fig. 6), including glycerophospholipid metabolism, sphingolipid metabolism, linoleic acid metabolism, alpha-Linolenic acid metabolism, glycerolipid metabolism, biosynthesis of unsaturated fatty acids, fatty acid degradation, arachidonic acid metabolism, purine metabolism. The most significantly enriched pathways included glycerophospholipid metabolism and sphingolipid metabolism, both of which are related to lipid metabolism. Further analysis revealed that the main metabolites contributing to the enrichment effects in these metabolic pathways still include LysoPC and PC.

#### Hierarchical Clustering Heatmap Analysis of Differentially Expressed Metabolites

**Fig. 7:**
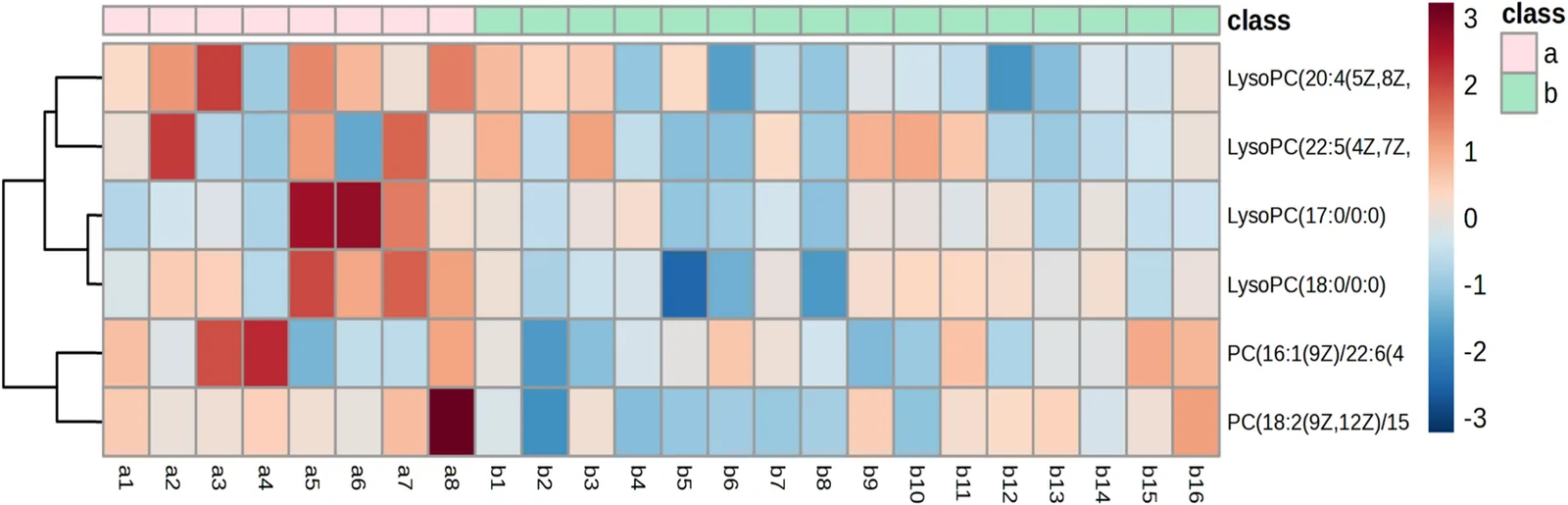
Heatmap of LysoPC and PC in hyperuricemic vs. normouricemic athletes at rest (a. hyperuricemic, b. normouricemic)

To visually illustrate the differences in metabolite abundances between hyperuricemic and normouricemic athletes, we performed hierarchical clustering heatmap analysis of relative intensities of differential serum metabolites using MetaboAnalyst 6.0. The heatmaps revealed simlar patterns for the two key metabolites: Compared with the control group, the hyperuricemia group showed a slight upward trend in LysoPC and PC levels, with a few samples exhibiting extremely high values (Fig. 7).

## Discussion

Recently, exercise physiology has increasingly adopted metabolomic technologies to profile endogenous compounds, identify exercise-related biomarkers, and elucidate metabolic pathways, leading to the emergence of exercise metabolomics [20]. In this study, untargeted metabolomics combined with PCA and OPLS-DA was used to characterize the serum metabolome across two stages: exercise stress and abnormal uric acid metabolism. We aimed to identify potential serum metabolite indicators associated with the early stages of uric acid alterations.

Pathway enrichment analysis revealed that both the high-intensity resistance exercise intervention and the hyperuricemia validation studies were significantly enriched in the glycerophospholipid metabolism pathway, with LysoPC and PC identified as the differential metabolites within this pathway. Glycerophospholipids are the most abundant compounds in the human body and essential components of biological membranes, directly participating in cell membrane signal transduction and protein recognition [21]. In glycerophospholipid metabolism, phosphatidylcholine (PC) can be hydrolyzed into lysophosphatidylcholine (LysoPC) by phospholipase A2 (PLA2); concurrently, in the presence of acyl-CoA, LysoPC can be reconverted into PC via lysophosphatidylcholine acyltransferase (LysoPCAT) [22]. The cycle between these two metabolites is a crucial aspect of lipid metabolism and a direct manifestation of cell membrane remodeling.

Following high-intensity resistance exercise, lipid metabolism in athletes becomes highly active, with PC hydrolyzed to LysoPC, resulting in an overall increasing trend of LysoPC and a decreasing trend of PC, indicating that cell membrane remodeling is in a rapid turnover phase [22]. Notably, correlation analysis between differential metabolites and clinical parameters further revealed the complex modulatory role of sex in this physiological process. Before adjusting for sex, changes in LysoPC(18:0) before and after exercise showed significant positive correlations with changes in UA, CK, urea, C, and T. Previous studies have demonstrated that high-intensity resistance exercise induces skeletal muscle microdamage, protein catabolism, and intense endocrine stress [23] and the substantial depletion of skeletal muscle ATP during strenuous exercise may accelerate purine nucleotide degradation, a potential underlying mechanism for exercise‑induced hyperuricemia [24]. LysoPC(18:0) may thus serve as a key lipid marker reflecting this stress‑induced metabolic state. However, after controlling for sex in partial correlation analysis, the correlations of LysoPC(18:0) with UA, CK, and cortisol weakened or even disappeared, with only a significant positive correlation with Urea remaining. This marked difference suggests that urea metabolism and related lipid profile alterations induced by high-intensity resistance exercise exhibit pronounced sexual dimorphism [25], which may be attributable to the larger muscle mass involved in males [26] and their higher baseline serum uric acid levels [27]. Furthermore, other lysophosphatidylcholine subspecies exhibited diverse metabolic trajectories: for example, LysoPC(P-16:0) showed significant negative correlations with urea and CK, suggesting that this LysoPC subspecies may be associated with a less pronounced muscle-injury-related metabolic response during exercise stress [28]; whereas the negative correlations of LysoPC(17:0) with TG and WBC, and the positive correlation of LysoPC(20:4) with WBC, further indicate that high-intensity resistance exercise may not only elevate uric acid through purine metabolism pathways but also involve a complex remodeling process characterized by LysoPC‑mediated immune‑inflammatory network activation [29] and systemic lipid mobilization.

In the hyperuricemia validation study, compared to normouricemic athletes, athletes with hyperuricemia exhibited an increasing trend in both LysoPC and PC at rest, with some samples showing pronounced elevations. Numerous lipidomic studies globally have consistently confirmed significant glycerophospholipid metabolism abnormalities in hyperuricemic patients [30–32], suggesting that hyperuricemia is not merely an imbalance of uric acid but may involve systemic lipid metabolism dysregulation. When exercise intensity exceeds an individual’s lipid metabolic capacity, it can induce physiological stress or even structural disruption [33]. Previous studies have found that the glycerophospholipid metabolic pathway can promote carnitine palmitoyltransferase 1 (CPT1)-mediated fatty acid oxidation, while simultaneously inhibiting glycolysis and enhancing oxidative phosphorylation. This leads to metabolic reprogramming and persistent immune system activation, inducing chronic inflammation that may impairs renal uric acid excretion by damaging renal tubular epithelial cells and downregulating uric acid transporter expression [34]. This provides a basis for how high-intensity resistance exercise might affect uric acid excretion via the glycerophospholipid pathway.

Currently, the prediction and diagnosis of hyperuricemia in athletes rely heavily on uric acid levels. However, when uric acid is significantly elevated, the body is often already in a state of metabolic stress. This study found that fluctuations in glycerophospholipids are closely correlated with changes in metabolic homeostasis. Accordingly, we hypothesize that the transient acute elevation of LysoPC following exercise primarily reflects a positive response to physiological stress and active lipid remodeling; whereas the persistently elevated LysoPC levels observed in hyperuricemic athletes at rest may indicate maladaptive long-term accumulation and a decline in the body’s “metabolic flexibility”.

This finding provides a specific molecular interpretation for the concept of metabolic flexibility in the context of exercise metabolism and hyperuricemia. Individuals with high metabolic flexibility can rapidly complete cell membrane repair and restore lipid metabolic function post-exercise, allowing lipid markers like LysoPC and PC to return to normal resting levels. Mouse experiments by Zhang et al. demonstrated that exercise remodels glycerophospholipid metabolism, with PC (18:1/18:1) mediating the anti-inflammatory effects of exercise, suggesting that exercise-induced downregulation of PC may be a hallmark of the body restoring homeostasis [35]. Conversely, in individuals with reduced metabolic flexibility, impaired lipid metabolic capacity leads to the accumulation of LysoPC and PC in the blood, manifesting as sustained lipid metabolism disorders. Long-term accumulation increases the risk of metabolic diseases such as hyperuricemia. In the resting state, highly active lipid metabolism in athletes is no longer a protective signal but may induce chronic inflammation and endothelial dysfunction, potentially suppressing renal uric acid excretion. Similar studies have also found significant positive correlations between total cholesterol (TC), triglycerides (TG), low-density lipoprotein (LDL), and uric acid, further indicating that hyperuricemia is inextricably linked to abnormal lipid metabolism [36]. Thus, LysoPC holds promise as a potential early-warning biomarker, as its aberrant fluctuations may precede a marked elevation in uric acid levels. Athletes with persistently high LysoPC levels during the resting phase, even if their uric acid levels have not yet met the clinical diagnostic criteria for hyperuricemia, should be considered at metabolic risk. Early interventions, such as adjusting training loads or implementing nutritional strategies, are necessary to restore their metabolic flexibility.

Although this study focuses on glycerophospholipid metabolism, previous research indicates that hyperuricemia is a systemic metabolic disorder. For instance, abnormal sphingolipid metabolism in hyperuricemic patients may also be closely related to post-exercise muscle damage repair and cell membrane homeostasis remodeling [21]. Other researchers focusing on amino acid metabolism in hyperuricemia have found that the pathogenesis of its complications involves not only uric acid elevation itself but also organ-specific reprogramming of amino acid metabolism [37]. Therefore, high-intensity resistance exercise can be viewed as a “metabolic stress test” [38] that reveals not only the limitations of an athlete’s individual lipid metabolic capacity but also the resilience and flexibility of their entire metabolic network. Dynamic monitoring of specific metabolite profiles before and after exercise may facilitate the early identification of individuals with slow recovery and suboptimal metabolic flexibility, thereby enabling proactive health management.

Of note, this study has limitations inherent to its self-controlled pre-post and cross-sectional design. Although a strong association between glycerophospholipid dysregulation and elevated uric acid levels was observed, a definitive causal relationship between the two cannot be established. Furthermore, the relatively small sample size of hyperuricemic athletes may affect the statistical power for analyzing certain differential metabolites. It is worth noting that in our cross-sectional validation cohort, the hyperuricemic group exhibited significantly higher body weight and BMI than the normouricemic group. This aligns with epidemiological evidence demonstrating a strong positive correlation between body weight (or BMI) and serum uric acid levels [39], particularly in weight-classified sports like judo where heavier athletes often face greater metabolic risks. While this weight difference reflects real-world clinical characteristics of hyperuricemic athletes, it may also act as a confounding factor for the observed metabolomic profiles. Future studies with strictly weight-matched cohorts are warranted to isolate the effects of uric acid from body weight on the metabolome. Most importantly, blood samples in this study were collected only within 10 min post-exercise, lacking consecutive monitoring data during the post-exercise recovery period (e.g., 2 h, 24 h, 48 h). Consequently, it is currently not possible to accurately distinguish whether the exercise-induced elevation of LysoPC represents a transient fluctuation that recovers within hours or a sustained accumulation that cannot be cleared, nor can we clarify individual differences in recovery rate or their specific relationship with the long-term risk of hyperuricemia. These hypotheses require future validation through large-scale, multicenter longitudinal cohort studies with multi-timepoint sampling, combined with targeted metabolomics techniques, to further evaluate the diagnostic performance of different LysoPC subspecies and to promote the clinical translation of these biomarkers.

## Conclusions

This study employed metabolomics technology to preliminarily reveal the metabolic network association between high-intensity resistance exercise stress and hyperuricemia. The results suggest that glycerophospholipid metabolism, specifically the interconversion between LysoPC and PC, plays a central role in the immediate metabolic response to exercise stress, thereby providing new molecular evidence for assessing the “metabolic flexibility” of the human body in response to exercise load. Aberrant glycerophospholipid metabolism may represent a potential metabolic link between acute exercise-induced stress responses and hyperuricemia-related metabolic profiles. LysoPC holds promise as a potential early-warning indicator for exercise-related uric acid metabolic disorders, offering a scientific reference for individualized training load monitoring and nutritional management in athletes. Future studies should expand cohort sizes, employ targeted metabolomics combined with long-term longitudinal follow-up designs, and include consecutive sampling time points during the recovery period to further clarify the causal relationship and clinical translational value between post-exercise lipid homeostasis recovery capacity and hyperuricemia.

## Data Availability

The datasets used and analysed during the current study are available from the corresponding author on reasonable request.

## Abbreviations

ALB: Albumin
C: Cortisol
Ca: Calcium
CHOL: Total cholesterol
CK: Creatine kinase
Cl: Chloride
CPT1: Carnitine palmitoyltransferase 1
ESI: Electrospray ionization
Fe: Serum iron
GLU: Glucose
Hct: Hematocrit
Hgb: Hemoglobin
HMDB: Human Metabolome Database
IGA: Immunoglobulin A
IGG: Immunoglobulin G
IGM: Immunoglobulin M
K: Potassium
LDL: Low-density lipoprotein
LysoPC: Lysophosphatidylcholines
LysoPCAT: Lysophosphatidylcholine acyltransferase
Mg: Magnesium
Na: Sodium
OPLS-DA: Orthogonal partial least squares discriminant analysis
P: Phosphorus
PC: Phosphatidylcholines
PCA: Principal component analysis
PLA2: Phospholipase A2
QC: Quality control
T: Testosterone
TC: Total cholesterol
TG: Triglycerides
UA: Uric acid
VIP: Variable importance in projection
WBC: White blood cell count
